# Individual and contextual factors associated with disposal of children’s stools in Papua New Guinea: evidence from the 2016–2018 demographic and health survey

**DOI:** 10.1186/s12889-020-09852-6

**Published:** 2020-11-23

**Authors:** Abdul-Aziz Seidu, Ebenezer Agbaglo, Bright Opoku Ahinkorah, Louis Kobina Dadzie, Ishmael Bukari, Edward Kwabena Ameyaw, Sanni Yaya

**Affiliations:** 1 Department of Population and Health, University of Cape Coast, Cape Coast, Ghana; 2 College of Public Health, Medical and Veterinary Sciences, James Cook University, Townsville, Queensland Australia; 3 Department of English, University of Cape Coast, Cape Coast, Ghana; 4 School of Public Health, Faculty of Health, University of Technology Sydney, Sydney, Australia; 5 Korle-Bu Teaching Hospital, Accra, Ghana; 6 School of International Development and Global Studies, University of Ottawa, Ottawa, Canada; 7 The George Institute for Global Health, The University of Oxford, Oxford, UK

**Keywords:** Disposal of children’s stools, Papua New Guinea, Public health, Sanitation, Socioeconomic status

## Abstract

**Background:**

Proper sanitation has been one of the topmost priorities on the global public health agenda. In the past few decades, sanitation programs targeting households have often paid little attention to the disposal of children’s stools. We assessed the individual and contextual factors associated with disposal of children’s faeces in Papua New Guinea.

**Methods:**

The data used for this study forms part of the 2016–2018 Papua New Guinea Demographic and Health Survey (PDHS). For this study, we focused on women with children less than five years (*n* = 2095). Both descriptive and inferential analyses were carried out. Descriptive statistics were used to summarize the data, using frequency counts and percentages. The inferential analysis used multilevel logistic regression models to investigate the individual and contextual factors associated with disposal of children’s stools. These models were presented as adjusted odds ratio (AORs), together with their corresponding 95% confidence intervals. Statistical significance was set at *p* < 0.05.

**Results:**

More than half (56%) of the women had disposed of their children’s stools unsafely. With the individual level factors, the results showed that women with children < 12 months [AOR =1.71; CI = 1.28–2.29] and women aged 20–24 [AOR =2.58; CI = 1.24–5.37], 35–39 [AOR =2.34; CI = 1.09–5.04], and 40 years and above [AOR =2.51; CI = 1.09–5.79] were more likely to practice unsafe disposal of children’s stool. The odds of unsafe disposal of faeces was also higher among women who visited the health facility for child diarrhea [AOR =1.69; CI = 1.25–2.28]. With the contextual factors, the odds of unsafe disposal of children’s stool was higher among women who lived in the Southern region [AOR =4.82; CI = 2.08–11.18], those who lived in male-headed households [AOR =1.79; CI = 1.19–2.70], and those who had unimproved toilet facilities [AOR =1.96; CI = 1.39–2.76]. On the contrary, women with unimproved source of drinking water were less likely to dispose of their children’s stool unsafely [AOR =0.54; CI = 0.35–0.83].

**Conclusion:**

Both individual and contextual factors predict unsafe disposal of children’s faeces in Papua New Guinea. It is recommended that sanitation programs should focus on behavioral change and not only on the extension of water and improved toilet facilities. Such programs should also focus on both individual and contextual factors of women.

## Background

As evident in the Sustainable Development Goal (SDG) six, proper sanitation has been one of the topmost priorities on the global public health agenda [1, 2]. This is because inadequate sanitation leads to the spread of disease-causing organisms through faeces and urine [3]. These organisms cause a variety of diseases, including cholera, diarrhoea, dysentery, hepatitis A, typhoid, and polio [4]. Globally, there are over 2.3 billion people without access to improved sanitation [5]. Research by Global Burden of Disease [6] pegged the global sanitation-related deaths at 775,000 annually. In low- and middle-income countries, about 5% of all deaths result from poor sanitation, while the global average is 1.4%. In the Pacific, about 70% of the population still use unimproved sanitation facilities, with 13% practicing open defecation [7]. The situation is worse in Papua New Guinea, where only 19% of the population have access to improved sanitation [7].

In the past few decades, sanitation programs targeting household sanitation have often paid little attention to the disposal of children’s stools. This results from the belief that children’s stools or faeces are less harmful compared to faeces of adults [8, 9]. However, that belief has been proven to be false, as evidence suggests that children’s stools can cause more serious faecal contamination in the household environment [10]. This is because, through some behaviour such as playing on the ground or crawling, children get their fingers exposed to faecal pathogens. Such children are also likely to put pica or fomite into their mouths [10]. It is, therefore, very important for every household to practice safe disposal of children’s faeces by putting the faeces into toilet/latrine or burying it. Practicices such as putting it in a drain or ditch, and throwing it in the garbage or in the open are considered unsafe practices [11].

Previous studies in India [12, 13], Bangladesh [14], Nigeria [15], Burkina Faso [16], and Ethiopia [17] have revealed positive associations between disposal of children’s stool, and individual/maternal and contextual/household factors. These studies have shown that women with high wealth status, those with higher levels of education [15, 16], women who live in urban areas [17], and those who have access to improved toilet facility [12, 17, 18] are more likely to safely dispose of children’s stool. These studies have provided adequate literature through their findings and recommendations which include strengthening policies that can increase caregivers’ awareness and practice of safe sanitation practices at all levels and in all livelihood domains [15, 16]. However, to the best of our knowledge, no study of this kind has been conducted in Papua New Guinea. In the present study, we sought to assess the individual and contextual factors associated with disposal of children’s stools in Papua New Guinea. This work will be beneficial to sanitation programs aimed at promoting safe disposal of children’s faeces.

## Methods

### Data source

The study was conducted in Papua New Guinea. According to the 2011 census report [19], the country has a total population of ﻿7,275,324 (﻿3,772,864 males and ﻿3,502,460 females). ﻿About 39% of the population live in the Highlands region, followed by Momase region, with 26%, while Southern and Island regions make up 20 and 15% respectively. According to the World Bank [20], as at 2019, about 86.8% of the population of Papua New Guinea were in rural areas. The data used for this study forms part of the 2016–2018 Papua New Guinea Demographic Health Survey (PDHS), which was collected from October 2016 to December 2018. The survey adopted a two-stage stratified sampling technique. Before the sampling, the provinces in the country were further apportioned into urban and rural areas, which yielded 43 strata; however, the National Capital District only had urban areas. A two-stage sampling procedure was used to sample census units (CUs) from each stratum. Stage One involved the selection of 800 CUs. The second stage saw the systematic selection of 24 households from each cluster through probability sampling, and this yielded a total of 19,200 households. The eligibility criteria for the interview included women of reproductive age (15–49) who were either regular members of selected households or slept in the household the night prior to the survey. The selected sample comprised 17,505 households. Of the total number of households selected, 16,754 were occupied, and 16,021 were successfully interviewed, with a response rate of 96%. Then, individual interviews were conducted on 18,175 women from the selected households, which yielded a response rate of 84%. Details of the methodology, pretesting, training of field workers, the sampling design, and selection are available in the PDHS final report available online at https://dhsprogram.com/publications/publication-fr364-dhs-final-reports.cfm. For this study, we focused on 2095 women with youngest children under five years who had complete information on all the variables of interest. We relied on the “Strengthening the Reporting of Observational Studies in Epidemiology” (STROBE) statement in conducting this study and writing the manuscript.

### Study variables

#### Outcome variable

The outcome variable was disposal of children’s stool, “safe/unsafe” [10, 12–15, 17, 21–23]. It was derived from the question, “The last time [Name] passed stools, what was done to dispose of the stools?” The responses were the following: “Child used the toilet or latrine,” “put/rinsed into toilet or latrine,” “put/rinsed into drain/ditch,” “thrown into the garbage,” “buried,” “left in the open,” and “other.” Following the WHO’s [11] definition of safe and unsafe stool disposal, these responses were recoded as follows: “child used toilet or latrine”, “buried” and “put/rinsed into toilet or latrine” were combined and coded as “safe disposal of child stool” (coded as ‘0’) whereas the others were coded as “unsafe disposal of child stool” (coded as ‘1’).

#### Independent variables

From the extensive literature review and availability of variables in the data, individual and contextual factors were considered as independent variables in this study. The individual level factors included age of child in months, sex of child, age of mother in years, mothers’ educational level, partners’ educational level, working status, frequency of reading newspapers, frequency of listening to radio, frequency of watching television, religion, and visit to health facility for child’s diarrhea treatment. The contextual variables were residence, region, sex of household head, source of drinking water, and type of toilet facility (see Table 1). These variables were included based on their association with disposal of children’s stool in previous studies [10, 12–15, 17, 21–23].

**Table 1:** Variables description and coding

Variable	Description	Coding
Child factors		
Child’s age in months	Age of child	1 = < 12 months 2 = 12-23 months 3 = 24+ months
Sex of child	Sex of child	1 = male 2 = female
Maternal factors		
Maternal age	Age of mother in years	1 = 15–19 2 = 20–24 3 = 25–29 4 = 30–34 5 = 35–39 6 = 40+
Mother’s educational level	Education level of mother	0 = No formal education 1 = Primary 2 = Secondary/higher
Partner’s educational level	Fathers educational level	0 = No formal education 1 = Primary 2 = Secondary/higher
Employment	Mothers employment status	1 = not working 2 = working
Frequency of reading newspaper or magazine	Do you read a newspaper or magazine at least once a week, less than once a week or not at all?	1 = Not at all 2 = Less than once a week 3 = At least once a week
Frequency of watching television	Do you watch television at least once a week, less than once a week or not at all?	1 = Not at all 2 = Less than once a week 3 = At least once a week
Frequency of listening to radio	﻿ Do you listen to the radio at least once a week, less than once a week or not at all?	1 = Not at all 2 = Less than once a week 3 = At least once a week
Religion	What is your religious affiliation?	1 = Orthodox 2 = Protestants 3 = Other
Visit to health facility for child diarrhea	In the past two months have you visited a health facility because your child had diarrhea?	1 = Yes 2 = No
Contextual factors		
Place of residence	Place of residence	1 = Urban 2 = Rural
Region	Region of residence	1 = Southern 2 = Highlands 3 = Momase 4 = Islands
Sex of household head	Sex of household head	1 = Male 2 = Female
Type of toilet facility	﻿What kind of toilet facility do members of your household usually use?	1 = Improved 2 = Unimproved
Source of drinking water	﻿What is the main source of drinking water for members of your household?	1 = Improved 2 = Unimproved

### Statistical analyses

The data were analysed with STATA version 14.2 for MacOS. Three basic steps were followed to analyse the data. The first step was the use of descriptive statistics to describe the sample (univariate analysis) and also tabulate all the independent variables against disposal of children’s faeces. The second step was a bivariate analysis using Pearson’s chi-square test of independence to select potential variables for the regression analysis. Variables that were statistically significant in the bivariate analysis at the *p* < 0.05 were retained. Afterwards, a three multilevel binary logistic regression analysis was done to assess the individual and contextual (household and community level) factors associated with disposal of children’s faeces. In this study, women and children were nested within clusters (primary sampling units), and clusters were nested within the regions. Clusters were considered as random effect to account for the unexplained variability at the regional level. We fitted four models (see Table 2). For all models, we presented the adjusted odds ratio (AOR) and associated 95% confidence intervals. For model comparison, we used the Akaike information criteria (AIC) test. We used the variance inflation factor (VIF) to test for multicollinearity, which showed no evidence of collinearity among the independent variables (Mean VIF = 1.13, Maximum VIF = 1.34 and Minimum VIF = 1.01). The svyset command was used to declare the survey data due to the complex sampling approach employed. The sample weight variable (v005/1,000,000) was applied in all the analyses to correct for over- and under-sampling of the respondents. All the reference categories for the logistic regression analysis were informed by previous studies [10, 12–15, 17, 21–23] and *a priori.*

**Table 2:** Disposal of children’s stool by demographic characteristics

Variable	Sample (***N*** = 2095)		Child stool Disposal practice		
	Weighted N	Weighted %	Safe(44%, CI = 42.9–45.9)	Unsafe (56%, CI = 54.5–58.0%)	*p*-value
**Child factors**					
**Child’s age in months**					*p* < 0.001
< 12 months	760	34.3	35.4	64.6	
12-23 months	628	30.0	52.9	47.2	
24+ months	**707**	33.8	45.8	54.2	
**Sex of child**					*p* = 0.948
Male	1125	53.7	45.35	54.65	
Female	970	46.3	42.74	57.26	
**Maternal factors**					
**Age of mother in years**					*p* < 0.01
15–19	58	2.9	45.62	54.38	
20–24	515	24.6	38.16	61.84	
25–29	624	29.8	44.77	55.23	
30–34	439	21.0	51.89	48.11	
35–39	304	14.5	43.12	56.88	
40+	154	7.4	40.91	59.09	
**Mother’s educational level**					*p* < 0.05
No education	619	29.5	37.40	62.60	
Primary	1030	49.2	45.45	54.55	
Secondary or higher	446	21.3	50.45	49.55	
**Partner’s educational level**					*p* = 0.29
No education	478	22.8	40.83	59.17	
Primary	869	41.5	45.33	54.67	
Secondary/higher	747	35.7	44.88	55.12	
**Working status**					*p* = 0.305
Not working	1438	68.6	44.31	55.69	
Working	657	31.4	43.76	56.24	
**Frequency of reading newspapers**					*p* = 0.269
Not at all	1564	74.7	42.73	57.27	
Less than once a week	302	14.4	52.57	47.43	
At least once a week	229	10.9	42.65	57.35	
**Frequency of listening to radio**					*p* = 0.526
Not at all	1466	70.0	45.55	54.45	
Less than once a week	363	17.3	38.95	61.05	
At least once a week	267	12.7	43.45	56.55	
**Frequency of watching television**					*p* = 0.139
Not at all	1733	82.7	45.77	54.23	
Less than once a week	172	8.2	39.18	60.82	
At least once a week	191	9.1	33.79	66.21	
**Religion**					*p* = 0.852
Orthodox	558	26.6	47.11	52.89	
Protestants	1108	52.9	42.62	57.38	
Other	429	20.5	44.20	55.80	
**Visit to health facility for child diarrhea**					*p* < 0.01
No	864	41.3	48.39	51.61	
Yes	1231	58.7	41.15	58.85	
**Contextual factors**					
**Place of residence**					*p* = 0.023
Urban	207	9.9	36.22	63.78	
Rural	1888	90.1	45.01	54.99	
**Region**					*p* < 0.001
Southern	432	20.6	35.11	64.89	
Highlands	739	35.3	43.01	56.99	
Momase	616	29.4	42.63	57.37	
Islands	308	14.7	62.55	37.45	
**Sex of household head**					*p* < 0.01
Male	1837	87.7	42.95	57.05	
Female	258	12.3	52.58	47.42	
**Type of toilet facility**					*p* < 0.01
Improved	570	27.2	42.77	57.23	
Unimproved	1525	72.8	44.65	55.35	
**Source of drinking water**					*p* < 0.001
Improved	403	19.3	31.68	68.32	
Unimproved	1692	80.8	47.11	52.89	

Source: PDHS (2016–2018)

## Results

### Disposal of children’s stool in Papua New Guinea

**Fig. 1:**
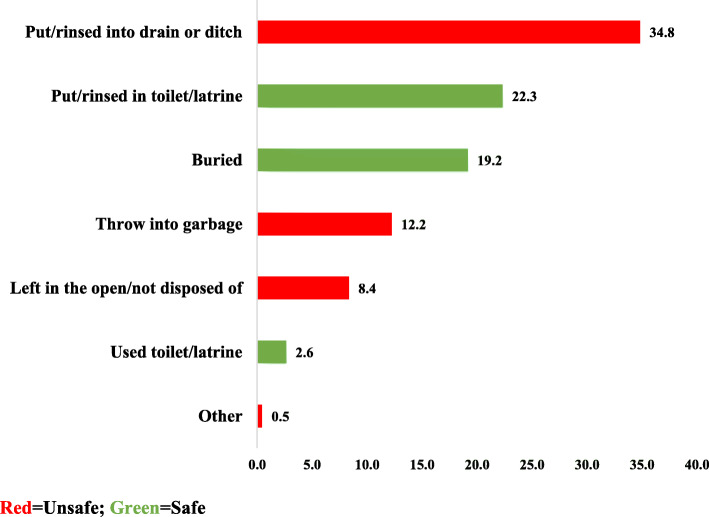
Prevalence of child faeces disposal practice in Papua New Guinea

It was found that more than half (56%) had disposed of their children’s stool unsafely while 44% disposed of their children’s stool safely (Table 2). For those who disposed of their children’s stool safely, 22.3% of them put/rinse into toilet/latrine while majority (34.8%) of those who disposed of their children’s stool unsafely put/rinse into a drain or ditch (see Fig. 1).

### Disposal of children’s stool by socioeconomic and demographic characteristics in Papua New Guinea

Table 2 shows the background characteristics of the women. It was found that 33.8% of the women had children aged 24+ months and 53% of the children were male. Approximately 30% (29.8%) of the women were aged 25–29, 49.2% had primary level of education, and 41.5% indicated that their partners also had primary level of education. We also noted that 68.6% of the women were not working. With access to mass media, 74.7%, 70%, and 82.7% indicated they were not exposed to newspaper, radio, and television respectively. More than half (52.9%) were Protestants and 58.7% visited the health facility for child diarrhea. Most of the women (90.1%) were in rural areas, 35.3% were in Highlands region, 87.7% lived in male-headed households, 72.8% had unimproved toilet facilities, and 80.8% had unimproved sources of drinking water. The results in Table 2 further showed that child’s age, mother’s age, mother’s educational level, visit to health facility for child diarrhea, place of residence, region, sex of household head, type of toilet facility, and source of drinking water had significant associations with disposal of children’s stool in Papua New Guinea.

### Factors associated with disposal practices of children’s stools in Papua New Guinea

#### Fixed effects results

Model IV of Table 3 shows results on the individual and contextual factors associated with disposal of children’s stool in Papua New Guinea. With the individual level factors, the results showed that women with children < 12 months were more likely to practice unsafe disposal of children’s stool, compared to those with children aged 24+ months [AOR =1.710; CI = 1.28–2.29, *p* < 0.05]. Unsafe disposal of children’s stool was also higher among women aged 20–24 [AOR =2.58; CI = 1.24–5.37 *p* < 0.05], 35–39 [AOR =2.340; CI = 1.09–5.04, *p* < 0.05], and 40 years and above [AOR =2.51; CI = 1.09–5.79, *p* < 0.05], compared to those aged 15–19. The odds of unsafe disposal of faeces was also higher among women who visited the health facility for childhood diarrhea, compared to those who did not visit [AOR =1.689; CI = 1.25–2.28, *p* < 0.05].

**Table 3:** Multilevel logistic regression of individual and contextual factors associated with disposal of children’s faeces in Papua New Guinea

Variable	Model I	Model II AOR [95% CI]	Model III AOR [95% CI]	Model IV AOR [95% CI]
**Fixed effects results**				
**Individual level factors**				
**Child’s age**				
< 12 months		1.67^***^[1.25,2.23]		1.71^***^[1.28,2.29]
12-23 months		0.84[0.63,1.12]		0.84[0.63,1.13]
24+ months		1		1
**Mother’s age**				
15–19		1		1
20–24		2.59^*^[1.25,5.37]		2.58^*^[1.24,5.37]
25–29		1.97[0.95,4.08]		1.94[0.93,4.03]
30–34		1.45[0.70,3.01]		1.43[0.68,2.98]
35–39		2.37^*^[1.10,5.08]		2.34^*^[1.09,5.04]
40+		2.74^*^[1.19,6.27]		2.51^*^[1.09,5.79]
**Mother’s education**al level				
No education		1.590^*^[1.02,2.48]		1.466[0.93,2.30]
Primary		1.457^*^[1.05,2.03]		1.344[0.96,1.89]
Secondary or higher		1		1
**Visit to health facility for child diarrhea**				
Yes		1.62^**^[1.20,2.18]		1.69^***^[1.25,2.28]
No		1		1
**Residence**				
Rural			0.75[0.39,1.42]	0.73[0.37,1.44]
Urban			1	1
**Region**				
Southern			4.94^***^[2.21,11.06]	4.82^***^[2.08,11.18]
Highlands			2.97^**^[1.34,6.58]	3.04^**^[1.32,6.99]
Momase			2.43^*^[1.04,5.69]	2.39[0.98,5.83]
Islands			1	1
**Sex of household head**				
Male			1.77^**^[1.19,2.64]	1.79^**^[1.19,2.70]
Female			Reference	Reference
**Type of toilet facility**				
Unimproved			1.94^***^[1.39,2.70]	1.96^***^[1.39,2.76]
Improved			1	1
**Source of drinking water**				
Unimproved			0.58^*^[0.38,0.88]	0.54^**^[0.35,0.83]
Improved			1	1
**Random effects results**				
**Parameters**				
Variance PSU	0.7088	0.76	0.35	0.400
Variance region	3.250	3.299	3.190	3.28
AIC	2546.1	2514.9	2516.3	2485.1
ICC PSU	0.098	0.103	0.051	0.057
ICC Region	0.546	0.552	0.519	0.528
Log-likelihood	− 1270.0	− 1244.4	− 1248.1	−1222.5
LR Test	354.5 (p < 0.001)	350.4 (p < 0.001)	283.9 (p < 0.001)	282.8 (p < 0.001)
Number of clusters	597	597	597	597
N	2095	2095	2095	2095

Exponentiated coefficients; 95% confidence intervals in brackets

^*^ *p* < 0.05, ^**^ *p* < 0.01, ^***^ *p* < 0.001, 1 = Reference category

*ICC* Intra-Class Correlation, *AIC* Akaike’s Information Criterion, *PSU* primary sampling units

Model 1 is the null model, a baseline model without any determinant variable;

Model II = individual level variables

Model III = Contextual Factors

Model III = Individual and Contextual Factors

With the contextual factors, the odds of unsafe disposal of children’s stool was higher among women who lived in the Southern region [AOR =4.82; CI = 2.08–11.18, *p* < 0.05], those who lived in male-headed households [AOR =1.792; CI = 1.19–2.70, *p* < 0.05], and those who had unimproved toilet facilities [AOR =1.961; CI = 1.39–2.76, p < 0.05], compared to those who lived in the Islands region, resided in female-headed households, and had improved toilet facilities. On the contrary, women with unimproved source of drinking water were less likely to dispose of their children’s stool unsafely [AOR =0.539; CI = 0.35–0.83, *p* < 0.05].

#### Random effects results

As shown in Model I, the clustering of the PSUs and region accounted for substantial variations in the odds of unsafe disposal of children’s stool (σ2 = 0.098 and 0.546, respectively). Model I showed that 9.8 and 54.6% of the total variation in unsafe disposal of children’s stool was attributed to the variance between the PSUs (ICC = 0.098) and region (ICC = 0.546). The between-cluster variance showed an increase from Model I to Model II (0.098 to 0.103). However, this decreased in both Models III (0.051) and IV (0.057). The same trend of ICCs was observed for variations in terms of region. This is a clear indication that the differences in unsafe disposal of children’s stool are mainly attributed to individual level factors. However, with a lowest AIC (2485.1) and a highest log-likelihood (− 1222.5), the best fit model is the final model (Model IV).

## Discussion

In the present study, we investigated factors associated with unsafe disposal of children’s stool in Papua New Guinea, with particular attention to mothers’ socioeconomic status. The study revealed that less than half (47%) of the study participants disposed of their children’s faeces safely. This suggests that more than half of the population may get their environments contaminated with children’s faeces, increasing the risk of human excreta which have adverse ramifications on origination and survival of disease-causing organisms [12]. The prevalence of safe disposal of children’s stool recorded in this study is higher than what was recorded in Madagascar (38%) [24] and Ethiopia (33.68) [17] but lower than what was found in Zambia (67%) [25], Kenya (70%) [26], Uganda (75%) [27], and Malawi (79%) [28]. The study revealed that, compared to women aged 15–19 years, all other women had higher odds of unsafe disposal of children’s faeces. All things being equal, women who are older than those in the 15–19 age category will have relatively much experience in birthing and wellbeing of children [29]. Due to this leverage in experience, these older women may underestimate the dire consequences of unsafe disposal of children’s faeces and rely on their personal experiences. Conversely, women aged 15–19 years are more likely to have had a single/first birth, with little experience in childcare, and as a result may adhere to the best practices and sanitation advice that they receive from healthcare providers.

Households with unimproved toilet facilities showed a higher likelihood of practicing safe disposal of children’s stool. This finding resonates with findings of Majorin et al. [12], Azage and Haile [17], and Sri and Puguh [18] in the context of India, Ethiopia, and Indonesia respectively. In relation to this finding, it has been argued that ownership of a latrine is a fundamental requirement for safe disposal of children’s faeces [12, 30]. This notwithstanding, nearly half of the households with improved toilet facility practiced unsafe disposal of children’s stool, suggesting that the availability of improved toilet facility does not guarantee safe disposal of children’s faeces. Some previous studies [12, 13, 31] made a similar observation. Allied with these studies is our finding that households with unimproved water supply have lower likelihood of disposing of children’s faeces unsafely. This suggests that the availability of improved water source alone is not sufficient to guarantee safe disposal of children’s faeces [17]. Plausibly, women who obtain water from unimproved sources are less motivated to practice unsafe faecal disposal as a strategy to reduce the susceptibility of their children to diarrhea and other poor sanitation-induced health conditions. This finding, however, runs contrary to what was reported by Oluko et al. [15], Curtis et al. [16], and Preeti et al. [13] in Nigeria, Burkina Faso, and India respectively.

Regional variations in unsafe disposal of children’s faeces were noted. Compared with Islands, those in Southern, Highlands, and Momase had higher odds of practicing unsafe disposal of children’s faeces. This points to the need for context-specific behavioural communication change interventions that can persuade all women of childbearing age to appreciate the need to always dispose of children’s faeces safely. When all women appreciate the graveness of the implications associated with unsafe disposal of children’s faeces, they would strive to ensure that all children’s faeces are safely disposed of to ensure good health for themselves and their children.

Women from male-headed households had higher odds of unsafe disposal of children’s faeces, compared with women from female-headed households. This finding suggests that female household heads possibly share their past experiences regarding children’s faeces disposal with women in the reproductive age in their households. Contrary to this, a study from India revealed that persons from male-headed households had higher chances of safe disposal of children’s faeces [32]. Contextual variations may account for the dissimilar findings.

Our study also revealed that age of children is associated with safe disposal of children’s faeces in Papua New Guinea. Specifically, women with children aged 12 months or younger showed higher likelihood of disposing of the faeces of their children unsafely, compared to those with children more than 12 months old. This confirms the findings reported in Bangladesh [14, 33]. This finding could be explained within the context of some misconceptions. For instance, there is a wrong belief that faeces of younger children are less harmful, relative to those of older children [8]. Similarly, there is the misconception that faeces of young children are smaller, have minimal stench, and contain fewer visible food residues, compared to faeces of older children which are believed to have bad smell and contain visible food residues which make them disgusting [34].

Finally, our study revealed an association between attendance to health facilities for child’s diarrhea and unsafe disposal of children’s faeces. What this means is that women who attended health facilities when their children had diarrhea recorded higher odds of practicing unsafe disposal of children’s faeces. In line with this, Horwood and Greenhill [33] have noted that enteric diseases cause majority of deaths in Papua New Guinea, adding that diarrhoea is among the commonest diseases that send people to health clinics and results in about 15% of deaths of children under five years of age. On the other hand, the finding suggests that being frequent at a health facility does not necessarily guarantee safe disposal of children’s faeces [35]. It is, therefore, imperative for healthcare providers to educate and remind women about the importance of safe disposal of children’s faeces anytime women report to the health facility to seek healthcare for any childhood illness.

### Strengths and limitations

The use of nationally-representative data with a relatively large sample size is the major strength of this study. The findings can, therefore, be generalized to all women in Papua New Guinea. Cause-effect relationship with reference to time i.e. temporality (not causality) cannot be ascertained since DHS was a cross-sectional survey. There is also the possibility of social desirability and recall biases. Finally, we acknowledge that there is the likelihood that some important factors (such as hand washing and soap use) may be excluded because they were not in the dataset. These are important factors in diarrhea transmission. It is, therefore, prudent to include these variables in future studies.

### Policy implications

The results from the study have revealed that there is relatively low prevalence of safe disposal of children’s faeces in Papua New Guinea. There are both individual and contextual factors associated with disposal of children’s stools. It is recommended that various policies and programs aimed at improving safe disposal of children’s faeces should focus on both individual and contextual factors. Specifically, sanitation programs should focus on behavioral change and not only on the extension of water and improved toilet facilities.

## Conclusion

The individual level factors associated with disposal of children’s faeces are child’s age, maternal age, and women visiting health facility for child diarrhea while the contextual factors are region of residence, sex of household head, type of toilet facility, and source of drinking water. Further studies could assess the association between the availability of hand hygiene resources (presence of water and soap for handwashing) and disposal of children’s stools.

## Data Availability

The dataset can be accessed at https:// https://dhsprogram.com/data/dataset/Papua-New-Guinea_Standard-DHS_2017.cfm?flag=0

## Abbreviations

AOR: Adjusted Odds Ratio
CI: Confidence Interval
PDHS: Papua New Guinea Demographic and Health Survey
WHO: World Health Organization
